# Excitonic and electronic transitions in Me–Sb_2_Se_3_ structures

**DOI:** 10.3762/bjnano.11.89

**Published:** 2020-07-16

**Authors:** Nicolae N Syrbu, Victor V Zalamai, Ivan G Stamov, Stepan I Beril

**Affiliations:** 1 Laboratory of Micro-Optoelectronics, Technical University of Moldova, 168 Stefan cel Mare Avenue, 2004 Chisinau, Republic of Moldova; 2 National Center for Materials Study and Testing, Technical University of Moldova, Bv. Stefan cel Mare 168, Chisinau 2004, Republic of Moldova; 3 T.G. Shevchenko State University of Pridnestrovie, 25 Oktyabrya street 107, 3300 Tiraspol, Republic of Moldova

**Keywords:** anisotropy, antimony triselenide, band structure, excitons, optical spectroscopy, reflection and absorption spectra

## Abstract

The optical anisotropy of the Sb_2_Se_3_ crystals was investigated at 300 and 11 K. Excitonic features of four excitons (A, B, C, and D) were observed in the optical spectra of the Sb_2_Se_3_ single crystals and in the photoelectric spectra of the Me–Sb_2_Se_3_ structures. The exciton parameters, such as the ground (*n* = 1) and excited (*n* = 2) state positions and the binding energy (Ry), were determined. The effective mass of the electrons at the bottom of the conduction band (*m*_c_* = 0.67*m*_0_) as well as the holes at the four top valence bands (*m*_v1_* = 3.32*m*_0_, *m*_v2_* = 3.83*m*_0_, *m*_v3_* = 3.23*m*_0_ and *m*_v4_* = 3.23*m*_0_) were calculated in the Г-point of the Brillouin zone. The magnitude of the valence band splitting V_1_–V_2_ due to the spin–orbit interaction (Δ_so_ = 35 meV) and the crystal field (Δ_cf_ = 13 meV) were estimated in the Brillouin zone center. The energy splitting between the bands V_3_–V_4_ was 191 meV. The identified features were discussed based on both the theoretically calculated energy band structure and the excitonic band symmetry in the Brillouin zone (*k* = 0) for crystals with an orthorhombic symmetry (*Рnma*). The photoelectric properties of the Me–Sb_2_S_3_ structures were investigated in the spectral range 1–1.8 eV under *E*||c and *E*⟂c polarization conditions and at different applied voltages.

## Introduction

Antimony selenide (Sb_2_Se_3_) is an inorganic semiconductor compound with interesting photoelectric properties. This material has a high absorption coefficient (≈10^5^ cm^−1^) in the region of maximum solar energy radiation [1–2] which is corroborated by a 6.5% rapid increase in solar cell efficiency when Sb_2_Se_3_ is present [3–5]. Interestingly, this high absorption coefficient is 10^3^ times higher than the absorption in silicon [5–7] and encompasses a wide portion of the spectrum ranging from 1.0 eV to 2–3 eV. The crystalline structure of Sb_2_Se_3_ is quite uniform and stable which minimizes the energy loss due to radiation [3,7–8]. In combination, the binary arrangement (Sb, Se), high crystalline stability, low toxicity and low deposition temperature (melting point ≈611 °С) reduce the production costs [3–10]. It has been shown that Sb_2_Se_3_ has many applications in photovoltaic devices and thermoelectric systems where it can be used as a thin film [11], in thermovoltaic and switch devices [12], in optical data storage [13] and in optoelectronics as a 2D anisotropic material [14–15].

In order to use Sb_2_Se_3_ to build high-performance devices it is necessary to study its crystalline nanostructure in terms of band structure and optical and optoelectronic properties, especially in the bandgap region in which ambiguous and contradictory results have been obtained. For example, the energy range of the bandgap was found to be 1.2 eV [15–16], 1.1–1.3 eV [17–18] and 1.25–1.46 eV [19] and these discrepancies have been pointed out in a different study [20]. There are also discrepancies in terms of which type of electronic transitions are responsible for determining the minimal bandgap. Several studies have shown that the bandgap is established due to allowed transitions that happen within 1.0 and 1.9 eV [6,8,10], whereas other studies show that the bandgap is determined by forbidden transitions [21–23]. In addition, the energy band structure and the theoretical calculations in the Brillouin zone space are also ambiguous [6,8,10,19–20].

The crystalline properties of Sb_2_Sе_3_, such as optical absorption, reflection, and photoconductivity, were studied in this work. In order to determine the bandgap, the nature of electronic transitions, among other properties, the absorption, reflection and excitonic spectra were obtained. The Sb_2_Se_3_ crystalline anisotropy of the ground and excited states of four excitonic series were determined at 300 and 11 K.

Due to the crystal field (Δ_cf_) and spin–orbit (Δ_so_) interactions, the high valence band splittings were estimated in the Brillouin zone center. The effective mass of the electrons and holes was calculated as well as the anisotropy of the latter. The photoconductivity measurements were performed in the excitonic region at positive and negative voltages applied to the Me–Sb_2_Se_3_ contacts. A similar investigation using the Sb_2_S_3_ single crystals was carried out by our group [24]. Since Sb_2_S_3_ and Sb_2_Se_3_ have a similar band structure, the four excitonic states (A, B, C and D) were also obtained for the Sb_2_S_3_ single crystals. Based in our previous work [24], the exciton binding energies, valence band parameters, valence band splitting, as well as the effective mass of electrons and holes were estimated for Sb_2_Se_3_ single crystals.

## Experimental

Bulk Sb_2_Se_3_ crystals were obtained by fusion (*T* ≈ 700–730 °C) of antimony (Sb) and selenium (Se) taken in the stoichiometric ratio. The growth method used for Sb_2_S_3_ [24] was adapted here for lower temperatures. Sb and Se, at a semiconductor purity В5 level (99.9999%), were used as the initial precursors and placed into a container that was evacuated to a residual pressure of 10^−5^ mmHg. For a thorough mixing of the reacting components in the liquid phase, a rocking device and an electromagnetic vibrator, at a frequency of *f* = 2 Hz, were used and the reaction lasted between six and eight hours. The ampoule with the synthesized material was placed in a temperature-gradient furnace. The synthesized Sb_2_Se_3_ was placed in the highest temperature zone of the furnace (720–730 °C) whereas the other end the of ampoule was designated as the crystal growth zone (670–680 °C). The ampoule was maintained at this temperature gradient for 80 h to allow for the crystal growth process. Due to the temperature gradient, the material was transferred to the crystal growth zone, which was set at the lower temperature range. The temperature difference between the two zones was approximately 50–60 °C which enabled single-crystalline growth. Easily-cleaved crystal ingots (1 × 1 × 1.5 cm) were the final product, from which mirrored layers of various thicknesses (100 μm–3 mm), were obtained. Thinner layers (1.3–10 μm) could also be obtained from the crystal with the aid of adhesive tape. X-ray diffraction was performed in order to verify the quality of the crystalline sheets and their spatial crystalline groups.

Optical transmission and reflection spectra were obtained on a double-grating spectrometer SDL-1 with a 1:2 aperture and 7 Å/mm linear dispersion. The crystals were placed in a closed helium cryostat LTS-22 C 330 perpendicular to the *b* axis and their spectra were obtained at low temperatures with ≈0.5 meV resolution since both the spectrometer entrance and exit slits did not exceed 70 µm. The crystal layers were characterized by a high reflectance, which is characteristic of metallic aluminum mirrors. Some measurements were also carried out on the spectrometer DFS-32 coupled with a Specord M-40 and a Jasco V-670. The photoconductivity spectra were obtained on a single spectrometer (MDR-2) with a 1:2 aperture and 7 Å/mm linear dispersion.

## Results and Discussion

**Figure 1:**
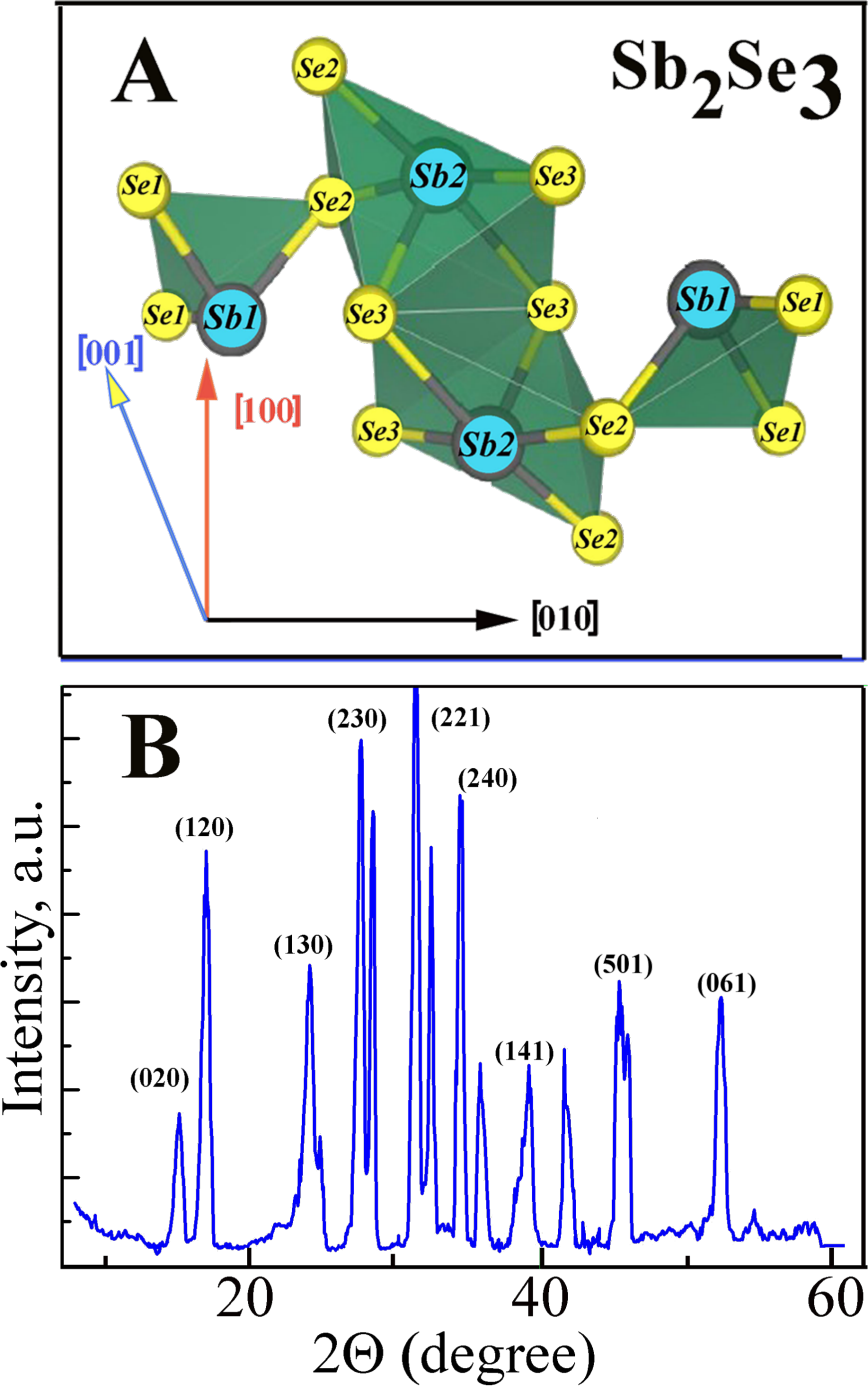
(A) Positions of the Sb and Se atoms in the Sb_2_Se_3_ crystals. (B) The Sb_2_Se_3_ crystalline XRD pattern.

The quality and composition of the single crystals were verified by optical and X-ray diffraction (XRD) analysis. The position of the atoms relative to the crystal lattice axes and the crystal XRD pattern is shown in Figure 1. A typical Sb_2_Se_3_ diffractogram is shown in Figure 1B. This result indicates the complete miscibility of the components during the synthesis process. The Sb_2_Se_3_ lattice parameters were determined based on the XRD analysis. The experimental interplanar distances *dhkl*, obtained from the X-ray data for Sb_2_Se_3_, are consistent with the previously published data [11]. The analysis shows that the prepared Sb_2_Se_3_ crystals are single phase and have an orthorhombic-type structure with a *Pnma* space group (*a* = 11.6901, *b* = 3.9210, *c* = 11.4894 Å) [21,25]. According to Figure 1A, which shows a fragment of the Sb_2_S_3_ crystal lattice, Sb_2_Se_3_ is a 2D semiconductor with a layered structure in which the Sb and Se atoms are connected with three other atoms of the opposite type, which in turn are connected within the crystal through weak secondary bonds.

**Figure 2:**
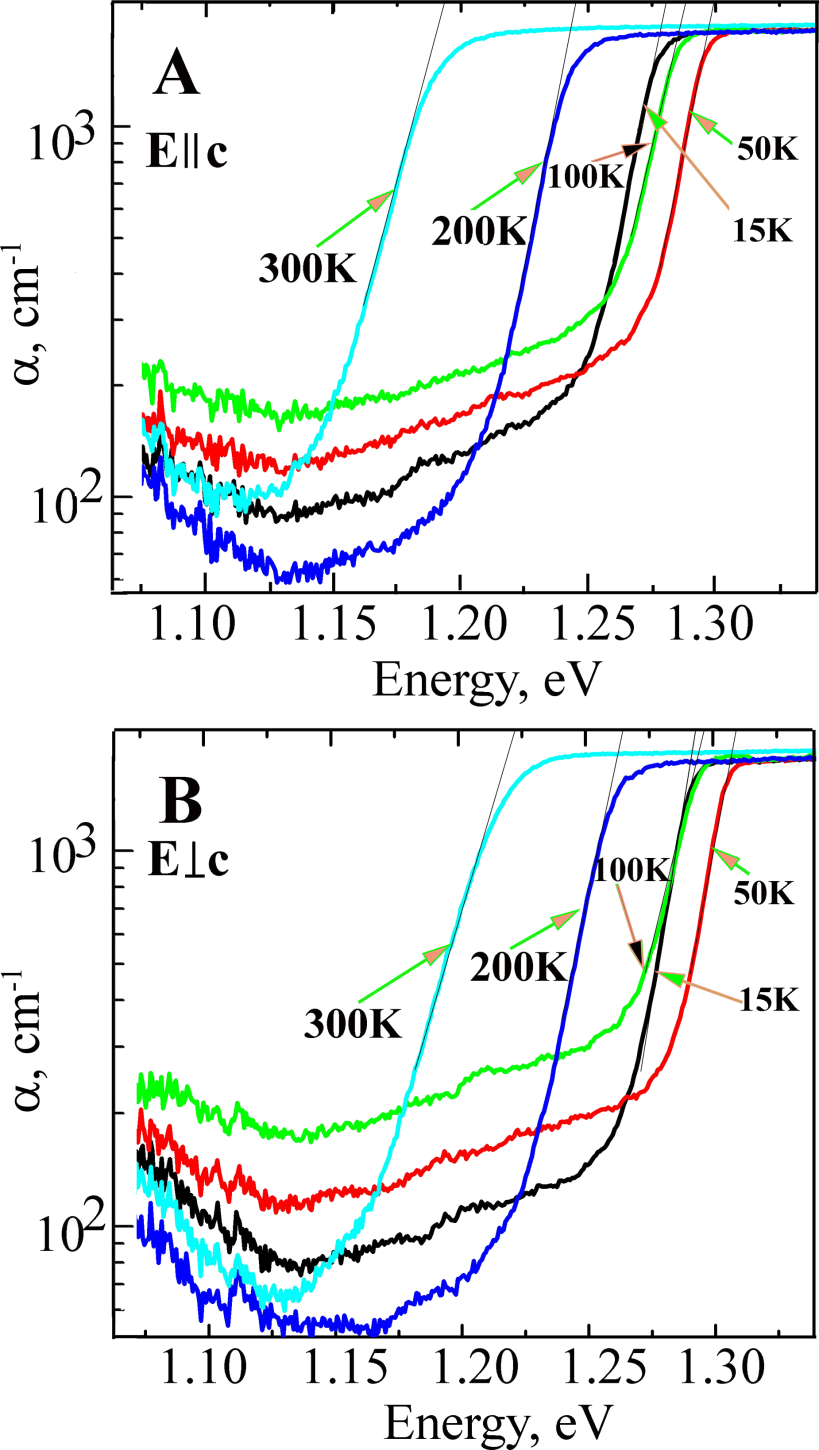
The absorption spectra of the Sb_2_Se_3_ crystals, with thickness *d* = 113 µm, measured at different temperatures (300 K: cyan, 200 K: blue, 100 K: green, 50 K: red and 15 K: black) for *Е*||с (A) and *Е*⟂с (B) polarization conditions.

Figure 2 shows the absorption spectra of the Sb_2_Se_3_ crystal (thickness *d* = 113 μm) measured at different temperatures (300–11 K) under *Е||*с and *Е*⟂с polarization conditions. The results demonstrate that the absorption edge is shifted towards higher energies when the temperature decreases. The largest difference in the absorption edge (*Е*_ed_) values is observed at ≈2·10^3^ cm^−1^ and at 300 K (Δ*Е* = *Е*_ed_(*Е*⟂с) – *Е*_ed_(*Е*||с) = 29 meV). When the temperature decreases to 100 K, Δ*E* decreases to 9 meV; however, a further decrease in the temperature to 11 K leads to an increase in Δ*E* to 16 meV (Table 1). Such absorption characteristics suggest that the absorption edge, under these polarization conditions, is formed due to the electronic transitions from different valence bands to a conduction band. Estimated values for the edge positions can be obtained by extrapolating the absorption curve to the energy axis, as shown by the black dotted lines in Figure 2.

**Table 1:** The differences (Δ*E*) in the absorption edge positions (*E*_ed_) for *Е*||с (*E*_ed_(*E*⟂c)) and *Е*⟂с (*E*_ed_(*E*||c)) polarization conditions at different temperatures.

*Т*, K	*Е*_ed_, *E*⟂c, eV	*E*_ed_, *E*||c, eV	Δ*E*, meV

300	1.220	1.191	29
200	1.264	1.244	20
100	1.296	1.287	9
50	1.307	1.296	11
11	1.294	1.278	16

**Figure 3:**
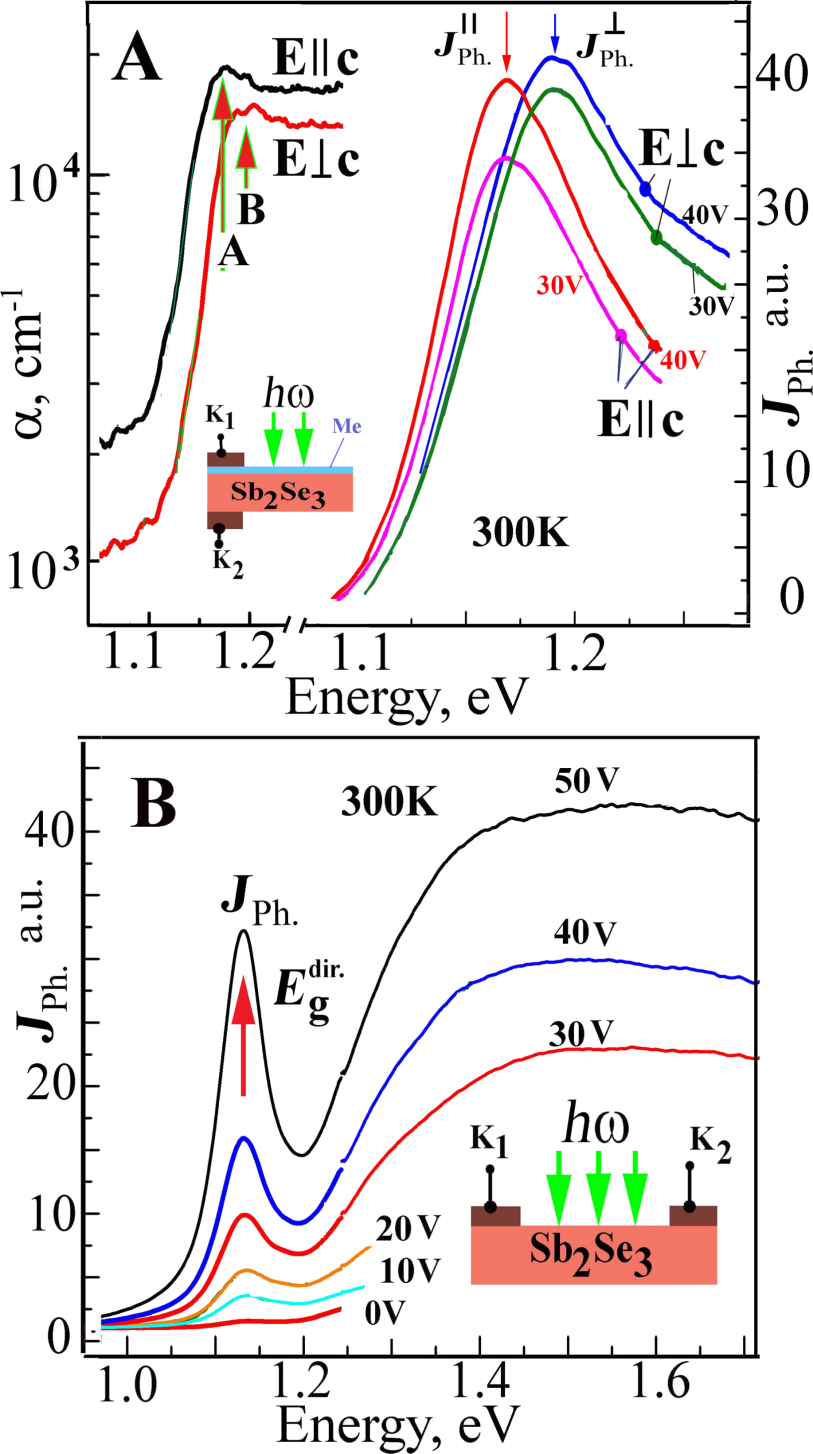
(A) The spectra showing the edge absorption (α) and photoconductivity (*J*_Ph_) for the Sb_2_Se_3_ crystals measured under *E*||c and *E*⟂c polarization conditions and at room temperature (insert shows the structure used for the photoconductivity measurements). (B) The photoconductivity spectra measured at room temperature for various voltages applied to the In–Sb_2_Se_3_ contact (insert illustrates this structure).

Figure 3A illustrates the absorption spectra of the Sb_2_Se_3_ single crystal with a 13 μm thickness measured at room temperature under *E||*c and *E*⟂c polarization conditions. The spectra show the high absorption coefficients with maxima at 1.162 eV (marked as A, *E||*c) and 1.185 eV (marked as B, *E*⟂c) at the absorption level of 2 × 10^4^ cm^−1^. The onset of edge absorption starts at 1.09 eV (*E||*c) and 1.1 (*E*⟂c). In conclusion, the absorption edge splitting (with a high absorption coefficient of ≈10^4^ cm^−1^) measured at room temperature is 23 meV.

In order to investigate the electrical and photoelectric properties of the metal–antimony selenide (In–Sb_2_Se_3_) contacts, the structures were obtained by either thermal sputtering under vacuum or electrochemical deposition onto the cleaved faces of single crystals (Figure 3A). Current–voltage characteristics suggest that the contacts have an ohmic behavior. The impedance has a frequency dependence that is characteristic of the conductivity hopping mechanism which in turn is independent of the metal type and the deposition method used. The photocurrent increases when the energy of the photons increases in the Schottky barriers when the transparent contacts are illuminated. The structures with the contacts deposited onto one side of the crystal are photosensitive. At the same time, the contact deposition onto opposite sides of the crystal planes leads to the appearance of a photo-electromotive force (EMF) with a magnitude of up to 150 mV. The photocurrent increases when a positive voltage is applied to the illuminated electrode and it decreases to zero when a negative voltage is applied. The nature of the photoelectric effect cannot be associated with the contact-EMF effect since there is no band bending at the semiconductor surface region. On the other hand, the photo-EMF effect, in this case, may be associated with the Dember effect.

The photoconductivity and photo-EMF spectra in the absorption edge region show a broad band with maximum values at 1.187 eV (*Е*⟂с) and 1.167 eV (*Е||*с) (Figure 3A) which are associated with the light absorption at the direct transitions in the interband gap minimum region. It is highly likely that the photoconductivity maxima are due to excitonic ground states (*n* = 1) in the aforementioned polarization conditions. In addition, the photocurrent increases when the bias increases. For the unpolarized light case, the photoconductivity spectra have a narrow maximum at 1.15 eV when different voltages are applied to the K_1_ and K_2_ contacts belonging to the structure shown in the Figure 3B insert. The maximum intensity increases when the voltage between the contacts increase which can be associated with the electronic transitions in the absorption spectra at 1.17 eV and 1.19 eV. When the applied voltage increases the photocurrent signal also increases in the region of higher energies (1.2–1.8 eV), reaching a maximum at 1.5–1.6 eV. The maximum photocurrent value at 1.15 eV is due to excitonic states at the direct electronic transitions between V_1_–C_1_ bands. The increase in intensity with the applied voltage confirms the excitonic character of the maximum, which is consistent with the fact that the binding energy of these excitons is 130–136 meV.

**Figure 4:**
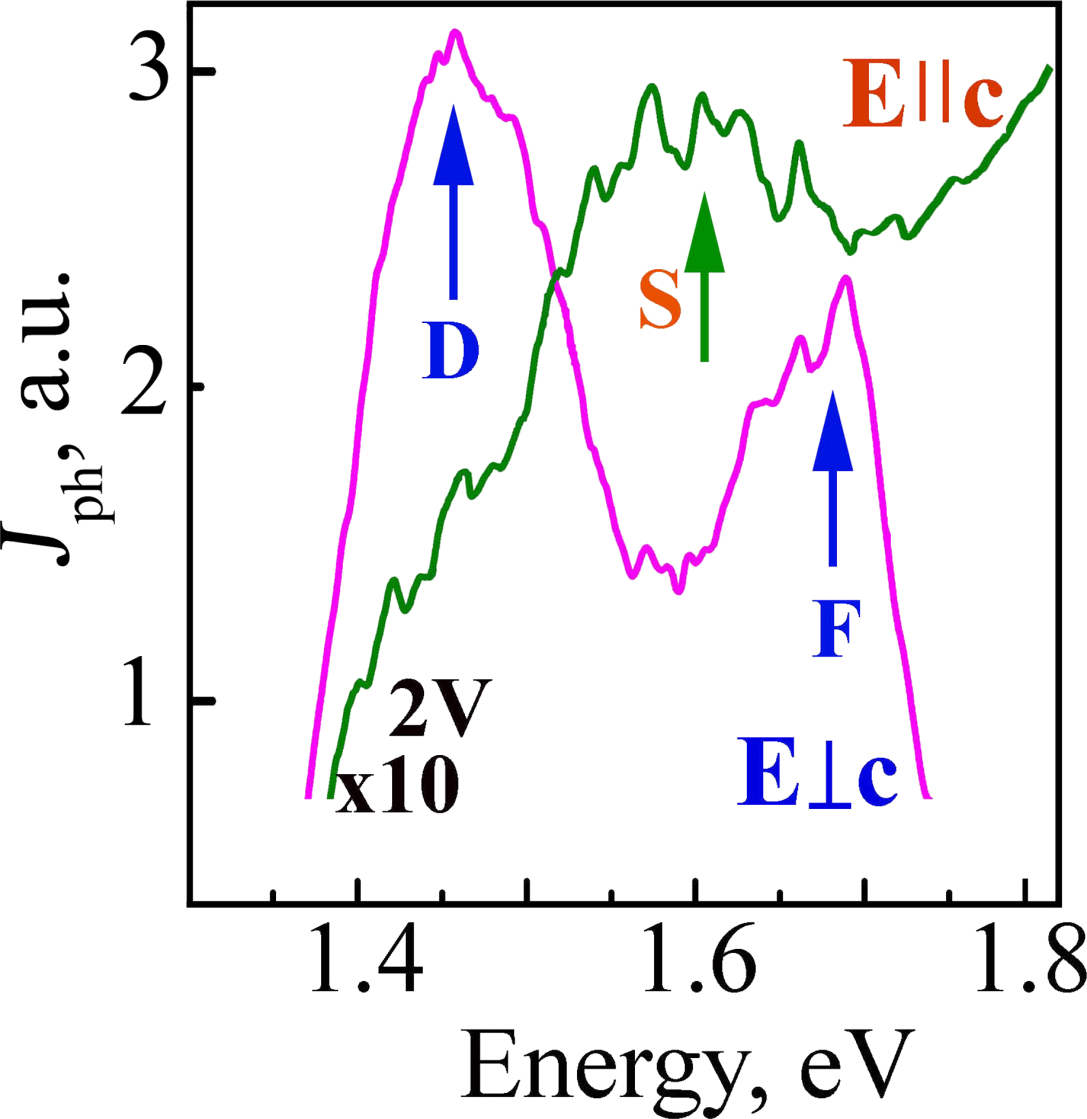
The photoconductivity spectra registered when 2 V is applied to the In–Sb_2_Se_3_ contacts (Figure 3A, insert) under the *E||*c and *E*⟂c polarization conditions.

The In–Sb_2_Se_3_ structures, in which the contacts were deposited by electrochemical methods, show photosensitivity over a wide energy range (1–1.8 eV, Figure 4). When a voltage range from 0 to 2 V was applied to the indium contacts, the photoconductivity maximum was registered at 1.168 eV for both polarization cases (*E*⟂c and *E||*c). This happens due to the direct electronic transitions in the bandgap minimum. The maxima D at 1.46 eV and F at 1.67 eV are observed in the photoconductivity spectra at the energy range between 1.3–1.8 eV for the *E*⟂c polarization case (Figure 4). For the *E||*c polarization condition, a lower maximum S is observed at 1.60 eV. All these maxima can be attributed to the direct electronic transitions in the Brillouin zone.

**Figure 5:**
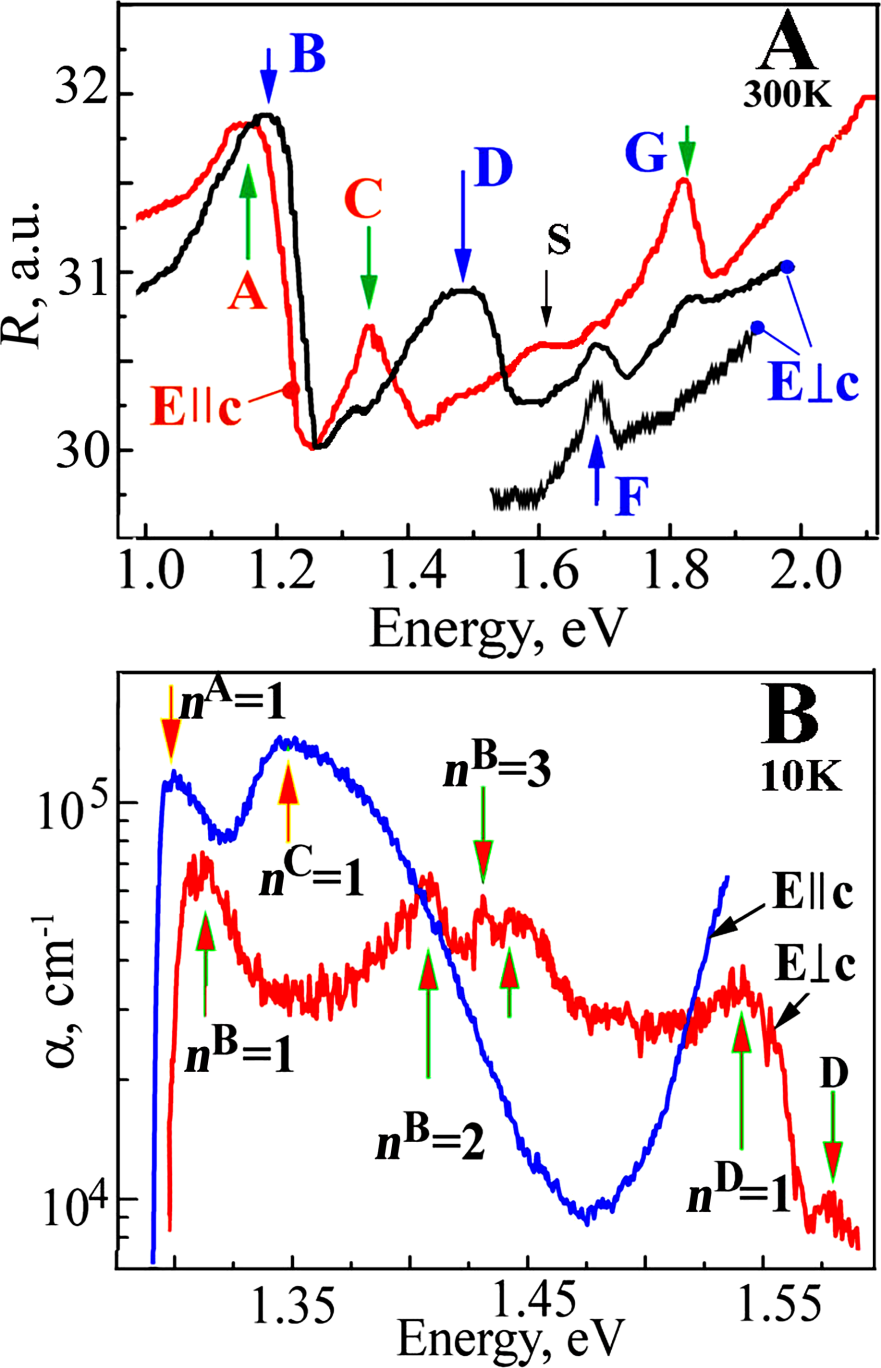
(A) The reflection spectra of the Sb_2_Se_3_ crystal measured at room temperature for both *E*||c and *E*⟂c polarization conditions. (B) The edge absorption of the Sb_2_Sе_3_ crystal with a thickness *d* = 1.3 μm for *Е*||с and *Е*⟂с polarization conditions at a temperature of 10 K.

The maxima observed for the reflection spectra (room temperature, *E||*c) are at 1.164 eV and 1.325 eV due to the ground states ***n***^A^ = 1 and ***n***^C^ = 1 for the A and C excitonic series, respectively. For the *Е*⟂с case, the reflection spectra maxima are at 1.191 eV and 1.467 eV due to the ground states for B and D excitons, respectively. A weaker shoulder S at 1.6 eV and a maximum G at 1.807 eV are measured for the *E||*c polarization case at higher energies. For *E*⟂c the polarization maximum F is at 1.671 eV (Figure 5A). There is good agreement between the maxima in the reflection (Figure 5) and in the photoconductivity spectra (Figure 4); therefore, these values can be attributed to the direct excitonic state transitions in the Brillouin zone.

The excitonic nature of the maxima detected in the reflection spectra is also confirmed by the absorption spectra measurements performed at low temperatures (Figure 5B). For the *Е||*c case, when the temperature decreases to 10 K, a maximum is detected at 1.299 eV which is caused by the exciton ground states ***n***^A^ = 1, conventionally designated as the A series. For the same polarization case, another maximum ***n***^С^ = 1 is detected at 1.347 eV, which is caused by the ground state of the C excitonic series. For the *E*⟂c polarization case six maxima are detected. In the long-wavelength region, a maximum is detected at 1.312 eV, which is caused by the B series. At 1.410 eV and 1.429 eV, the excited states ***n***^B^ = 2 and ***n***^B^ = 3 of the B excitonic series are observed. In the high-energy region, the maximum ***n***^D^ = 1 is detected at 1.538 eV and a weaker peak is detected at 1.588 eV, which is formed by the D excitonic series in the vicinity of another pair of bands. To determine the main parameters of the excitonic series, the profiles of the measured reflection spectra of the A and B excitons (experimental data) are calculated based on the dispersion ratios in the single-oscillator and multi-oscillator models, according to a method described in our previous work [26].

**Figure 6:**
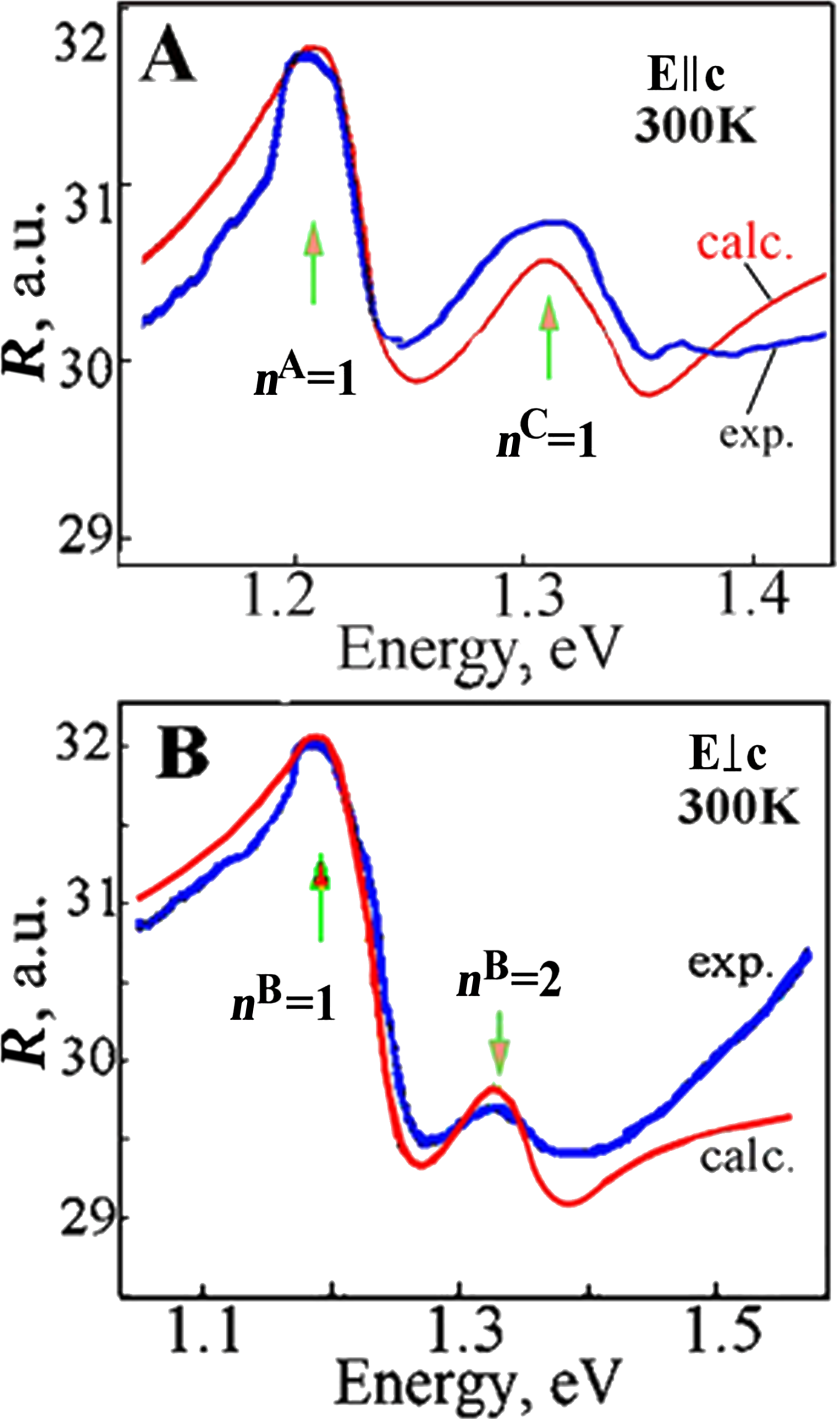
The experimentally measured (exp.) and calculated (calc.) profiles of the reflection spectra for the *Е*||с (A) and *Е*⟂с (B) polarization cases at 300 K.

Figure 6 shows the experimentally measured and the calculated profiles of the reflection spectra for both *Е||*с and *Е*⟂с polarization cases at 300 K. The calculations showed that for the polarization *Е||*с the background dielectric constant (ε_b_) is equal to 7.5, the energy of the transversal exciton (ω_Т_) is 1.192 eV, the longitudinal-transversal splitting (ω_LT_) is 15 meV, the damping factor (γ) is 110 and the translational mass of the exciton (*M*) is 3.5*m*_0_ (Table 2). For the excitonic series С the following parameters were calculated: ω_Т_ = 1.310 eV, ω_LT_ = 17 meV, γ = 150 and *M* = 3.9*m*_0_. For the *E*⟂c polarization case the calculations of the reflection spectra profiles gave the following parameters: ε_b_ = 7.5, ω_Т_ = 1.219 eV, ω_LT_ = 14 meV, γ = 161, and *M* = 4.5*m*_0_.

**Table 2:** Exciton parameters of the Sb_2_Se_3_ crystals (data outside brackets: from the reflection spectra, data inside brackets: from the absorption spectra).

Exciton state	*E*||c, A-exc.	*E*⟂c, B-exc.	*E*⟂c, C-exc.	*E*⟂c, D-exc.
	*R*, 300 K/ (α, 11 K)	*R*, 300 K/ (α, 11 K)	*R*, 300 K/ (α,11 K)	*R*, 300 K/ (α, 11 K)

*n* = 1, eV	1.164/ (1.299)	1.191/ (1.312)	1.310/ (1.347)	1.522/ (1.538)
*n* = 2, eV	–	1.317/ (1.410)	1.372	/ (1.588)
*n* = 3, eV	–	(1.429)	–	–
ω_LT,_ meV	15.0	14.0	17.0	–
Ry, eV	–	0.168/ (0.130)	0.082	0.067
*E*_g_, eV	–	1.359/ (1.442)	1.392	1.589
ε_b_	7.5	7.5	–	8.5
μ*, *m*_0_	0.56	0.56	–	0.49
*M*, *m*_0_	3.5	4.5	3.9	3.9
*m*_c_^*^, *m*_0_	0.67	0.67	0.67	0.67
*m*_v1_^*^, *m*_0_	3.32	–	–	–
*m*_v2_^*^, *m*_0_	–	3.83	–	–
*m*_v3_^*^, *m*_0_	–	–	3.23	–
*m*_v4_^*^, *m*_0_	–	–	–	3.32

By using the obtained experimental data and the known relation 
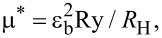
 where *R*_H_ is the Rydberg energy of a hydrogen atom (13.6 eV) and Ry is the binding energy for the corresponding exciton (Rydberg constant), the reduced effective mass (μ*) is calculated for the excitons A, B, C and D. For excitons A and B when the background dielectric constant is ε_b_ = 7.5 and the binding energy is Ry = 130–136 meV the reduced exciton mass is μ* = 0.56*m*_0_. For the exciton series C at ε_b_ = 7.5 and at the binding energy Ry = 82 meV, the reduced mass of the exciton is μ* = 0.49*m*_0_. The Bohr radius (α_B_) for the S state of the A exciton is 0.3 × 10^–5^ cm and for the B exciton it is α_B_ = 0.2 × 10^–5^ cm. Considering that the exciton mass *M* is equal to the sum of the masses of holes and electrons, *m*_v_* + *m*_c_*, and the reduced mass 1/µ* is equal to (1/*m*_v_*) + (1/*m*_c_*), from the experimentally estimated mass values of *M* and µ*, the effective mass is estimated for the electrons in the conduction band *m*_c_* = 0.67*m*_0_ and for the holes in the valence bands *m*_v1_* = 3.32*m*_0_, *m*_v2_* = 3.83*m*_0_, *m*_v3_* = 3.23*m*_0_ and *m*_v4_* = 3.32*m*_0_ (Table 2). The excitonic parameters calculated here correlate with the previously published data [18], where it was stated that Frenkel excitons exist in the Sb_2_S_3_ crystals with a binding energy Ry = 0.1 eV and effective mass *m*_c_* = 1.035*m*_0_, *m*_v1_* = 1.843*m*_0_. A similar approach that was used in our previous work to study the Sn_2_S_3_ crystals was also used here for the calculation of the effective mass of electrons and holes in the bands located at the Brillouin zone center. The magnitudes of the effective mass of the electrons (*m*_c_^*^ = 1.08*m*_0_) at the bottom of the conduction band and of the holes at the top of four valence bands (*m*_v1_^*^, *m*_v2_^*^ = 2.91*m*_0_ and *m*_v3_^*^, *m*_v4_^*^ = 3.12*m*_0_) were estimated [24]. The bandgap was calculated based on the positions of the ground and excited states of the observed excitons. The well-known formula *E*_g_ = *E*_i_ + Ry/*n*^2^ was used for this calculation, where *E*_g_ is the bandgap energy, *E*_i_ corresponds to the positions of the ground (*n* = 1) and excited (*n* = 2, 3, 4…) states of the exciton, Ry is the exciton binding energy (Rydberg constant) and *n* = 1, 2, 3 … are the main quantum numbers. First, from the positions of the ground and excited states, the Rydberg constant was calculated. Then the bandgap energy is estimated.

**Figure 7:**
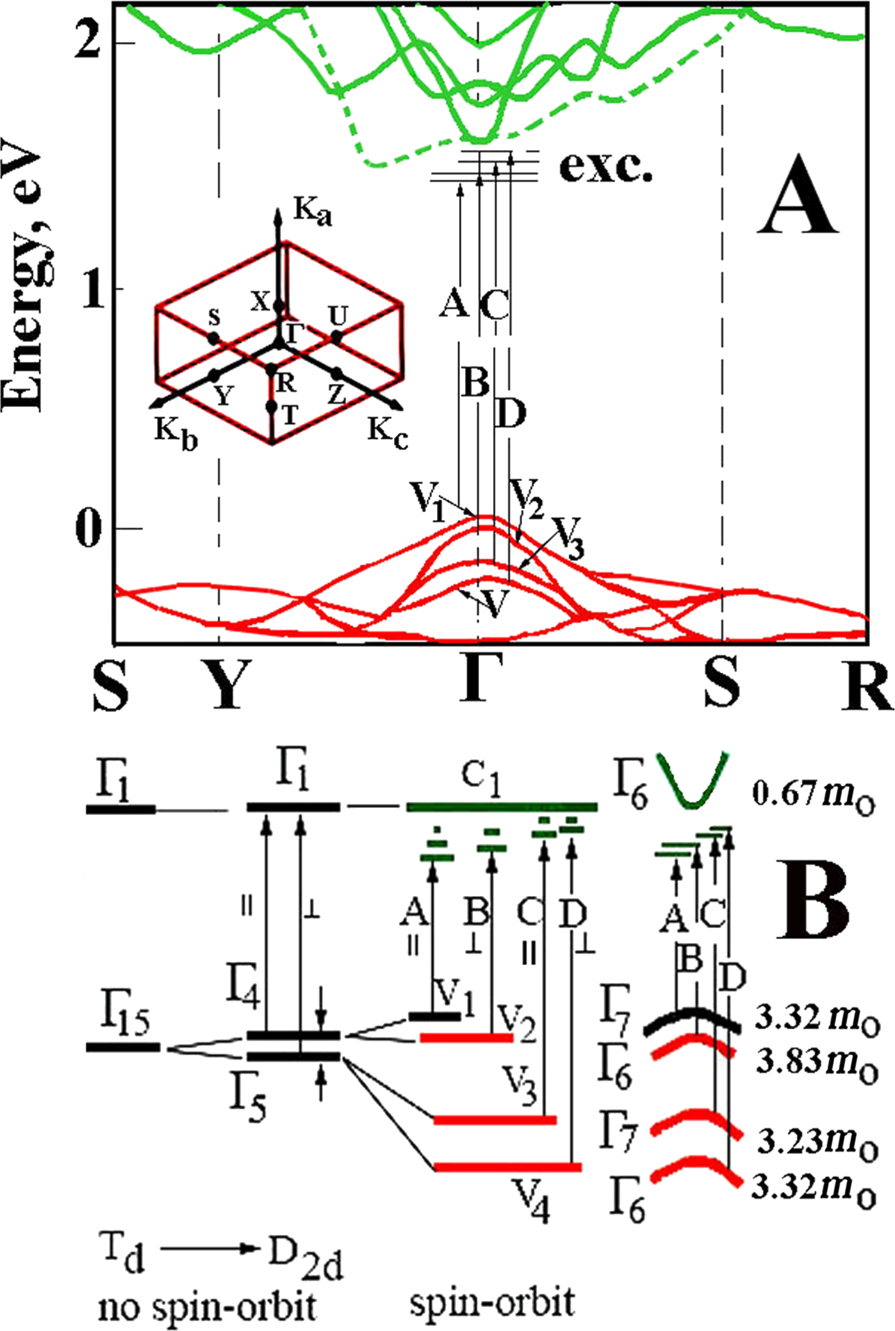
(A) The energy band structure of the Sb_2_Sе_3_ crystals. Insert illustrates the Brillouin zone. (B) Transformation from the tetrahedral to orthorhombic symmetry due to the spin–orbit interaction and the crystal field in the center of the Brillouin zone. Figure 7B adapted with permission from [24], copyright 2020 Elsevier.

In the Sb_2_Se_3_ and Sb_2_S_3_ crystals, the theoretical calculation of the band structure over a wide energy range was performed in several studies [6,8,10,19]; however, the obtained results were contradictory and the inconsistencies were related to the assignment of the actual points in the Brillouin zone. For all the previous studies [6,8,10,19] the valence bands had the maximum in the Brillouin zone center (in *k* = 0, Г-point) whereas the minimum in the conduction band was found to be in different points of the Brillouin zone. For example, a few studies [8,10,19] showed that the minimum was localized in the Z point whereas others [6] found the minimum in the X point. In addition, for the Sb_2_S_3_ crystals [20], the minimum energy interval corresponded to direct transitions in the center of the Brillouin zone (Г-point). For the Sb_2_Se_3_ crystals the top of the valence band was positioned between the Γ and S points, while the bottom of the conduction band was in the Γ point. Given the inconsistency in the literature, our results were discussed based on theoretical calculations performed by Koç and collaborators [20]. A similar approach was used to interpret our previous data for the Sb_2_S_3_ crystals [24]. Based on the results by Koç et al. [20], Figure 7 illustrates the band structure fragment in the interband minimum region. As mentioned previously, Koç and collaborators [20] calculated and built a wavevector space for both crystals (Sb_2_S_3_ and Sb_2_Se_3_) band structures. Based on these data an interpretation was made in terms of the electron transitions in the framework of the calculated band structure. As mentioned, a few previous studies have shown that the absorption edge was formed by indirect transitions in Sb_2_Se_3_. However, the data presented here does not confirm entirely the previous findings and it does not refute the existence of indirect transitions given that more studies (like the ones made for Ge, Si, GaP, etc.) need to be performed in order to clarify those issues, ideally with purely grown crystals. We do not negate the existence of indirect transitions as we do not have such experimental data. Therefore, a focus will be given on the direct transitions that were experimentally observed (Figure 7).

The excitonic series A, B, C, and D is formed by the electrons in the conduction band *C*_1_ (with Г_6_ symmetry) and the holes in the valence bands V_1_, V_2_, V_3_, and V_4_ (with Г_7_, Г_6_, Г_7_, and Г_6_ symmetries), respectively [27]. Considering that the bands originate from the structures with a higher symmetry (tetragonal) to the structures with an orthorhombic symmetry (*D*_2_*_h_*), it should be noted that the bands in *k* = 0 are split by a crystal field and a spin–orbit interaction [27]. The lower conduction band is formed from the Г_1_ states and acquires the Г_6_ (Г_7_) symmetry, while the upper valence bands of V_1_, V_2_, V_3_, and V_4_ have the Г_7_, Г_6_, Г_7_, and Г_6_ symmetry, respectively. The interaction between the electrons from the Г_6_ conduction band and the holes from the Г_7_ valence band is determined by the product of the irreducible representation Г_1_ × Г_6_ × Г_7_ = Г_3_ + Г_4_ + Г_5_. As a result of this interaction, in the long-wavelength region, an exciton Г_4_ is allowed in polarization *E||*c, Г_5_ is allowed in polarization *E*⟂c and Г_3_ is forbidden in both polarization conditions. The interaction between the electrons from the *C*_1_ conduction band (Г_6_ symmetry) with the holes from the V_2_ valence band (Г_6_ symmetry) causes the appearance of three excitonic series: Г_1_, Г_2_ and Г_5_. According to the selection rules for the *Е*⟂с polarization case, the Г_5_ excitons are allowed whereas the Г_1_ and Г_2_ excitons are both forbidden. A similar approach was used when the Sb_2_S_3_ single crystals were investigated [24]. Since the Sn_2_S_3_ and Sn_2_Se_3_ crystals have the same crystal structure and a similar band structure (only with a different bandgap) the excitons observed had the same symmetries. Based on the obtained experimental data, the splitting between the upper valence bands, V_1_ and V_2_, in the center of the Brillouin zone is 13 meV, whereas the splitting between V_2_ and V_3_ is 35 meV and between V_3_ and V_4_ is 191 meV.

**Figure 8:**
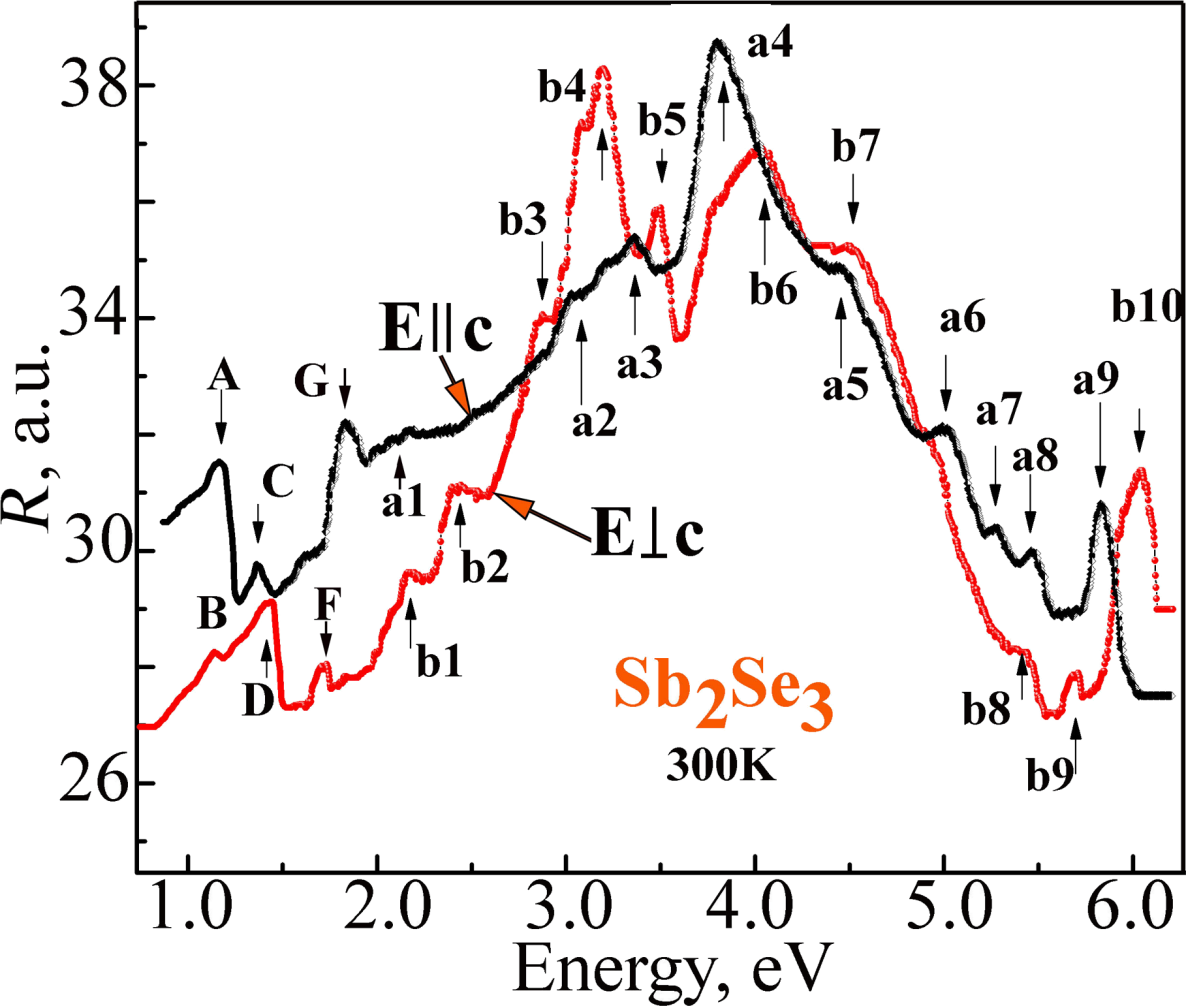
The reflection spectra of the Sb_2_Se_3_ crystals measured at room temperature under *Е*||с and *Е*⟂с polarization conditions.

Besides the excitonic peaks (A, B, C and D), the features a1 (2.090 eV), a2 (3.059 eV), a3 (3.365 eV), a4 (3.822 eV), a5 (4.432 eV), a6 (5.009 eV), a7 (5.281 eV), a8 (5.466 eV) and a9 (5.815 eV) are also observed in the reflection spectra (Figure 8). These spectra were measured at room temperature over a wide energy range (1–6 eV) under the *E||*c and *E*⟂c polarization conditions. The observed reflection peaks can be associated with the direct electronic transitions at actual points of the Brillouin zone. In the *Е*⟂c polarization, the reflection spectra maxima b1 (2.167 eV), b2 (2.439 eV), b3 (2.875 eV), b4 (3.191 eV), b5 (3.485 eV), b6 (4.040 eV), b7 (4.509 eV), b8 (5.391 eV), b9 (5.685 eV) and b10 (6.045 eV) can be identified. The maxima a1, b1, b2, and b3 are most likely due to the electronic transitions from the valence bands V_1_, V_2_, V_3_ and V_4_ to the conduction band C_2_ in the Brillouin zone center. The maxima localized in the high-energy region of the reflection spectra a2, a3, a4, a5, b4, b5, b6 and b7 are most likely associated with the transitions from the upper valence bands V_1_, V_2_, V_3_ and V_4_ to the conduction bands C_3_ and C_4_ also in *k* = 0. The maxima of the reflection spectra in the high-energy range (4–6 eV) are possibly from the valence band maxima in the Y and S points of the Brillouin zone.

## Conclusion

The ground and excited states of four excitonic series (A, B, C, and D) formed in the bandgap minimum region were identified based on the studies of the optical properties of the Sb_2_Se_3_ single crystals performed at different temperatures. Taking into account the energy position of the excitonic ground and excited states, the binding energy of the excitons and the valence bands V_1_–V_4_ were determined. In the Brillouin zone Г-point, the calculated electron effective mass *m*_c_* was 0.67*m*_0_, and the values of the hole effective masses *m*_v1_*, *m*_v2_*, *m*_v3_* and *m*_v4_* were 3.32*m*_0_, 3.83*m*_0_, 3.23*m*_0_ and 3.32*m*_0_, respectively. The V_1_–V_2_ valence band splitting in the center of the Brillouin zone by a crystal field (Δ_cr_ = 13 meV) and the spin–orbit interaction (Δ_so_ = 35 meV) were determined. The bands V_3_–V_4_ were split by 191 meV. The observed features were discussed based on the theoretical calculation of the energy band structures and the excitonic band symmetries in the Brillouin zone center for crystals with an orthorhombic symmetry (*Pnma*). The In–Sb_2_Se_3_ structures were generated either by thermal sputtering under vacuum or by electrochemical deposition. The photoconductivity spectra at different applied voltages were investigated. The features associated with the excitonic states were shown in the measured photoconductivity spectra.

## References

[1] Zhou Y, Wang L, Chen S, Qin S, Liu X, Chen J, Xue D-J, Luo M, Cao Y, Cheng Y (2015). Nat Photonics.

[2] Zeng K, Xue D-J, Tang J (2016). Semicond Sci Technol.

[3] Wang L, Li D-B, Li K, Chen C, Deng H-X, Gao L, Zhao Y, Jiang F, Li L, Huang F (2017). Nat Energy.

[4] Chen C, Wang L, Gao L, Nam D, Li D, Li K, Zhao Y, Ge C, Cheong H, Liu H (2017). ACS Energy Lett.

[5] Messina S, Nair M T S, Nair P K (2009). J Electrochem Soc.

[6] El-Shair H, Ibrahim A, Abd El-Wahabb E, Afify M, Abd El-Salam F (1991). Vacuum.

[7] Zhou Y, Leng M, Xia Z, Zhong J, Song H, Liu X, Yang B, Zhang J, Chen J, Zhou K (2014). Adv Energy Mater.

[8] Chen C, Li W, Zhou Y, Chen C, Luo M, Liu X, Zeng K, Yang B, Zhang C, Han J (2015). Appl Phys Lett.

[9] Voutsas G P, Papazoglou A G, Rentzeperis P J, Siapkas D (1985). Z Kristallogr.

[10] Mueller R, Wood C (1972). J Non-Cryst Solids.

[11] Ko T-Y, Shellaiah M, Sun K W (2016). Sci Rep.

[12] Fourspring P M, DePoy D M, Rahmlow T D, Lazo-Wasem J E, Gratrix E J (2006). Appl Opt.

[13] Arun P, Vedeshwar A G, Mehra N C (1999). J Phys D: Appl Phys.

[14] Song H, Li T, Zhang J, Zhou Y, Luo J, Chen C, Yang B, Ge C, Wu Y, Tang J (2017). Adv Mater (Weinheim, Ger).

[15] Kurumada M, Suzuki H, Kimura Y, Saito Y, Kaito C (2003). J Cryst Growth.

[16] Gilbert L R, Van Pelt B, Wood C (1974). J Phys Chem Solids.

[17] Kutasov V A (2005). Shifting the maximum figure-of-merit of (Bi, Sb)2(Te, Se)3 thermoelectrics to lower temperatures. Thermoelectrics Handbook.

[18] Liu X, Chen J, Luo M, Leng M, Xia Z, Zhou Y, Qin S, Xue D-J, Lv L, Huang H (2014). ACS Appl Mater Interfaces.

[19] Zhang Y, Li G, Zhang B, Zhang L (2004). Mater Lett.

[20] Koç H, Mamedov A M, Deligoz E, Ozisik H (2012). Solid State Sci.

[21] Černošková E, Todorov R, Černošek Z, Holubová J, Beneš L (2014). J Therm Anal Calorim.

[22] El-Sayad E A (2008). J Non-Cryst Solids.

[23] Vadapoo R, Krishnan S, Yilmaz H, Marin C (2011). Phys Status Solidi B.

[24] Beril S I, Stamov I G, Tiron A V, Zalamai V V, Syrbu N N (2020). Opt Mater.

[25] Green M A, Emery K, Hishikawa Y, Warta W, Dunlop E D, Levi D H, Ho-Baillie A W Y (2017). Prog Photovoltaics.

[26] Syrbu N N, Ursaki V V, Bergin R M (2010). Exciton polariton dispersion in multinary compounds. Exciton Quasiparticles: Theory, Dynamicsand Applications.

[27] Kovalev O V (1986). Irreducible and Induced Representations and Corepresentations of Fedorov Groups.

